# The EBV-Positive Tumor Methylome Is Distinct from EBV-Negative in Diffuse Large B-Cell Lymphoma

**DOI:** 10.3390/cancers17182994

**Published:** 2025-09-13

**Authors:** Ashley K. Volaric, Ramiro Barrantes-Reynolds, Karine Sahakyan, Yuri Fedoriw, Seth Frietze

**Affiliations:** 1 Department of Pathology and Laboratory Medicine, University of Vermont Larner College of Medicine, University of Vermont Cancer Center, Burlington, VT 05401, USA; 2 Department of Microbiology and Molecular Genetics, University of Vermont, Burlington, VT 05401, USA; 3 Department of Radiology, University of Vermont Larner College of Medicine, Burlington, VT 05401, USA; 4 Department of Pathology and Laboratory Medicine, University of North Carolina Chapel Hill School of Medicine, Chapel Hill, NC 27599, USA; 5 Department of Biomedical and Health Sciences, University of Vermont, University of Vermont Cancer Center, Burlington, VT 05401, USA

**Keywords:** Epstein–Barr virus, EBV, diffuse large B-cell lymphoma, DLBCL, methylation, methylome, epigenetics

## Abstract

**Backgrounds**: Epstein–Barr virus (EBV) is implicated in the pathogenesis of different B-cell lymphomas and lymphoproliferative disorders, including diffuse large B-cell lymphoma (DLBCL) arising in immunodeficiency settings. Despite its clinical significance, the mechanisms of EBV-mediated lymphomagenesis across different disease subtypes remain poorly understood. Global DNA methylation profiling can provide insight into tumor heterogeneity and disease mechanisms. **Methods**: To further characterize the underlying biology of EBV(+) DLBCL, we performed a global methylome analysis of a cohort of EBV(+)/(−) DLBCL. Illumina MethylationEPIC array data were generated from a curated set of DLBCL tissue samples (*n* = 43) from a rural patient population with defined EBV status and immunodeficiency background. Differential methylation analyses were conducted using linear mixed models to identify significant methylation changes associated with EBV status. **Results**: Principle component analysis (PCA) and probe-level comparisons revealed a distinct, globally hypermethylated DNA methylome in EBV(+) DLBCL compared to EBV(−) cases, and an overall hypomethylated profile in all DLBCL relative to control tissues. We identified a total of 117,334 differentially methylated probes mapping to 1557 cancer-associated genes in EBV(+) versus EBV(−) DLBCL, and 330,872 probes mapping to 4230 cancer-associated genes in all DLBCL versus controls. Pathway enrichment analysis highlighted distinct biological processes in EBV(+) DLBCL, including P53 feedback loops (hypermethylated genes) and MAPK signaling (hypomethylated genes). **Conclusions**: These findings demonstrate that EBV(+) DLBCL is epigenetically distinct from EBV(−) disease, with alterations that may contribute to clinical heterogeneity and potentially serve as biomarkers for disease classification and therapeutic targeting.

## Simple Summary

Epstein–Barr virus (EBV) is a ubiquitous virus associated with the development of B-cell lymphoma, including diffuse large B-cell lymphoma (DLBCL) arising in the setting of immunodeficiency and dysregulation. EBV-associated DLBCL is more clinically aggressive than EBV(−) disease, but targeted therapeutic options have not been developed. This is in large part due to the lack of understanding regarding disease pathogenesis mediated by the virus. One way the virus instigates lymphoma is through epigenetically modifying host B-cell DNA, promoting tumor formation and cellular transformation. Our study seeks to understand and characterize how EBV acts epigenetically in the host B-cell to promote DLBCL tumor formation. Using a high-throughput microarray technique that globally characterizes methylation status across the human genome (Illumina MethylationEPIC array), we found that EBV(+) DLBCL exhibits a unique DNA methylation environment distinct from EBV(−) DLBCL. This suggests that the virus functions at an epigenetic level to significantly alter genetic pathways and promote tumor cell formation. Additional studies are needed to further investigate the epigenetic mechanisms behind EBV-associated lymphoma development to identify possible therapeutic targets for this aggressive disease.

## 1. Introduction

Epstein–Barr virus (EBV)-driven diffuse large B-cell lymphoma (DLBCL) represents a clinically aggressive tumor subtype that is often refractory to standard R-CHOP chemotherapy (Rituximab, Cyclophosphamide, Doxorubicin Hydrochloride, Vincristine Sulfate, and Prednisone) [1,2,3]. Currently, no targeted treatment options exist for EBV(+) DLBCL. While EBV is generally accepted to play a role in the pathogenesis of DLBCL, the specific disease mechanisms and the significance of the viral contribution in vivo have not been clearly defined. Furthermore, no consensus standard exists for diagnosing DLBCL as EBV-positive using the conventional in situ hybridization tissue stain for EBV-encoded small RNA (EBER-ISH) [4,5,6,7,8,9]. Until EBV diagnostics and pathogenic mechanisms are elucidated, the development of targeted treatments for EBV(+) DLBCL will remain a challenge.

Understanding the role of EBV in modifying the epigenetic landscape of DLBCL offers promising avenues for understanding viral pathogenesis, identifying diagnostic biomarkers, and developing targeted therapeutics. In vitro studies using EBV-infected cell lines have shown that the virus directly modifies both host cellular epigenome and its own genome through DNA methylation [10,11]. DNA methylation allows for dynamic changes in gene expression via hypermethylation (silencing) or hypomethylation (activation) of functional promoter regions. These alterations serve as epigenetic “switches” that can respond to external environmental cues, such as those present in the immunosuppressed tumor microenvironment. In vitro evidence has demonstrated the capacity of EBV to modulate the host epigenome by upregulating oncogene expression and downregulating tumor suppressor genes, thereby promoting B-cell malignant transformation [10,11,12]. Our study conducted further investigation on in vivo clinical tissue samples of EBV(+)/(−) DLBCL to better define the tumor cell methylome and clarify the epigenetic role of EBV.

We characterized the DLBCL methylome in EBV-mediated disease using the high-throughput DNA methylation microarray Illumina MethylationEPIC v2.0, which interrogates over 935,000 CpG sites across the human genome. This approach has been successfully employed across numerous in vivo cancer studies, including The Cancer Genome Atlas (TCGA), which profiled thousands of samples across diverse tumor types [13,14,15,16]. The Illumina EPIC methylation array is particularly well-suited for formalin-fixed tissue, allowing for comprehensive genome-wide methylation profiling. Through this technology, the TCGA project has systematically characterized epigenomic alterations, yielding insight into oncogene activation, epigenetic regulation of gene expression, and tumor microenvironment. These insights have informed advances in diagnostics and targeted immunotherapies [14].

We applied the Illumina EPIC methylation array to a clinical cohort of EBV(+)/(−) DLBCL to identify distinct tumor methylomes associated with EBV-driven oncogenesis. This pilot study demonstrates that EBV(+) DLBCL harbors a unique signature compared to EBV(−) disease, underscoring the need for future investigation in larger cohorts.

## 2. Materials and Methods

### 2.1. Clinical Study Population and Tissue Sample Collection

A total of 31 clinical tissue cases of DLBCL from our institution, including 9 EBV(+) and 22 EBV(−), from 2000–2023 were selected for the study. In addition, 12 control tissue cases were selected over this period for comparison, including 2 EBV(+) polymorphic lymphoproliferative disorders, 2 EBV(+) plasmacytic hyperplasia post-transplant lymphoproliferative disorders, 4 EBV(+) infectious mononucleosis tonsil/lymph nodes, and 4 EBV(−) reactive follicular hyperplasia lymph nodes. The cases originated from a wide range of immunosuppressive states including post solid organ/stem cell transplantation, systemic chemoradiation therapy-related (iatrogenic), autoimmune disease treated with systemic immunosuppressive pharmacotherapy (e.g. methotrexate, long-term steroid use, etc.), and primary immunodeficiency. The contribution of immunosenescence, as defined by age >65 years old, was likely in most tumor cases.

Representative hematoxylin and eosin-stained (H&E) slides for each case were reviewed by the study hematopathologist (Volaric) to confirm diagnoses. Standardized automatic ISH staining platform (Leica Biosystems) for EBER-ISH was used in the evaluation. All cases were categorized as EBV(+) using EBER-ISH (ISH5687-A). EBV positivity was defined in DLBCL or control cases as exhibiting at least 50% cellular staining in either tumor cells or lymphocytes for EBER-ISH [5]. EBV-negative cases showed no cellular staining. All EBV(+) DLBCL cases were then characterized for latency status by immunohistochemistry (IHC) using commercially available antibodies for LMP-1 (CS 1–4, Abcam ab78113) and EBNA-2 (PE2, Abcam ab90543). Positive expression of LMP-1 and EBNA-2 was defined as ≥1% staining in tumor/lesional cells, and negative expression as no cellular staining. Latency state was categorized as the following: latency I (negative expression of LMP-1 and EBNA-2), latency II (positive LMP-1 and negative EBNA-2), and latency III (positive expression of both LMP-1 and EBNA-2). Only cases in which diagnostic material was obtainable (formalin-fixed paraffin-embedded (FFPE) tissue blocks or unstained sections) were included. The study was approved by the UVM Institutional Review Board.

### 2.2. DNA Methylation Analysis

#### 2.2.1. Global Methylation Profiling Using Illumina EPIC Methylation Array

FFPE tumor and control tissue sections were macro-dissected and sent to CD Genomics (NY, USA) for Illumina Infinium MethylationEPIC BeadChip array (935K). Samples were subjected to DNA extraction using protocols optimized for archival FFPE material. DNA quantity and quality were assessed prior to array hybridization. The array includes >935,000 CpG probes that span gene promoters, enhancers, gene bodies, and intergenic regions, and includes known cancer-associated loci and open chromatin regions. After hybridization and scanning, raw data were delivered in the form of IDAT files containing methylated and unmethylated signal intensities.

#### 2.2.2. Bioinformatic Analysis

Illumina EPIC methylation analysis was performed using R (version 4.4.1). Raw IDAT files were processed using the SeSAMe package (v1.22.2) [17]. Initial quality filtering was conducted using the SeSAMe pOOBAH (*p*-value with out-of-band [OOB] array hybridization) method, which performs dye bias correction and calculates detection *p*-values. The background subtraction was conducted using the QCDPB criteria: Q—qualityMask Masking probes of poor design; C—inferInfiniumIChannel Infer channel for Infinium-I probes (needed for dye bias correction step); D—dyeBiasNL actual dye bias correction (non-linear); P—pOOBAH Detection *p*-value masking using oob; B—noob background subtraction using oob. Only samples with median intensity >10.5 and mean detection *p*-values < 0.05 were retained (*n* = 43), resulting in 889,341 high-confidence probes for downstream analysis. Normalized beta values (ranging from 0 to 1) were used for downstream analysis. Principal component analysis (PCA) was performed using ‘prcomp()’ to assess global methylome clustering. Batch effects were tested through visual inspection of PCA plots as well as statistical association between the principal components and batch using linear regression. No batch effects were found. Differential methylation analysis was performed using linear models implemented in the ‘DML’ (differential methylation locus) function of the SeSAMe package, comparing EBV(+) vs. EBV(−) DLBCL and DLBCL vs. control tissues. Probe-level significance was adjusted using *p*-values of 0.05. Significant probes were annotated to genes using sesameAnno_attachManifest for EPICv2. Significant probes were aggregated at the gene level to identify differentially methylated regions (DMRs) based on a log2 fold change cutoff and multiple testing thresholds. For enrichment analysis of gene sets, we used the testEnrichment function of the SeSAMe package, which performs a Fisher’s exact test to determine if the number of significant probes associated with a gene is greater than expected by chance. We further used oncoEnrichR (v1.5.2) to map differentially methylated genes to curated cancer pathways. Immune cell composition inference employed IDOL Optimized CpGs from the Illumina Human Methylation Dataset package FlowSorted.Blood.EPIC (v2.8.0). This estimated the relative abundance of immune cell populations across samples. Differences in immune composition between EBV(+) and EBV(−) DLBCL were assessed using the Wilcoxon rank-sum test (*p* < 0.05). Commercially available antibodies were used for immunohistochemical analysis of CD20 (Ventana Ultra, Thermo Fisher L26), CD3 (Leica, Biocare LN10), and CD56 (Ventana Ultra, Roche MRQ-42) on a representative case of EBV(+) and EBV(−) DLBCL.

## 3. Results

### 3.1. Clinicopathologic Characteristics of Study Cohort

**Figure 1:**
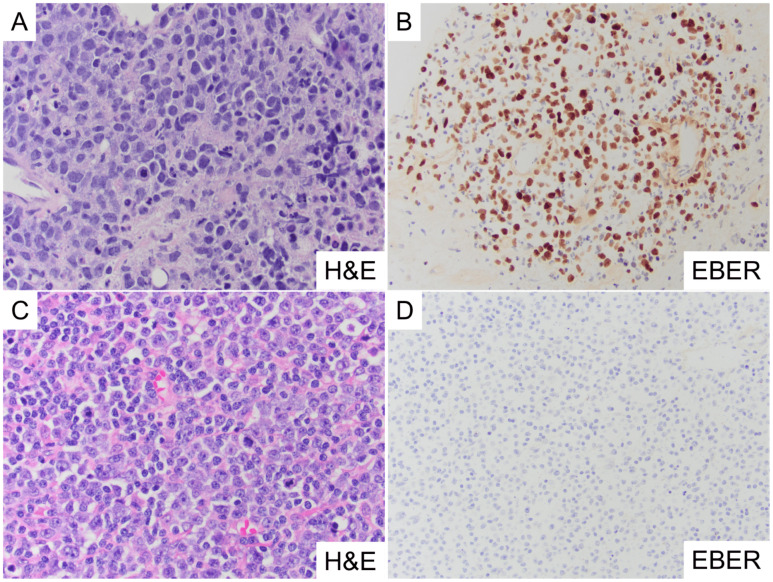
Representative histological images of EBV(+) and EBV(−) DLBCL. (**A**,**B**) EBV(+) DLBCL showing prominent large B-cells in a high-powered field with a mixed inflammatory background of small lymphocytes, granulocytes, and apoptotic debris (**A**, H&E, 40×). EBER in situ hybridization (ISH) highlighting >80% nuclear staining in tumor B-cells, marking an EBV(+) DLBCL. A lower-powered field was utilized to demonstrate the overall EBER staining pattern in the cellular population (**B**, EBER, 20×). (**C**,**D**) EBV(−) DLBCL composed predominantly of sheets of large B-cells with mitotic figures and apoptotic debris (**C**, H&E, 40×). EBER stain is negative for nuclear cellular staining in tumor cells, indicating an EBV(−) DLBCL (**D**, EBER, 20×).

To elucidate the DNA methylation profiles of EBV-mediated DLBCL, we established a clinical cohort of DLBCL (*n* = 43) from our institution, consisting of EBV(+) DLBCL (*n* = 9), EBV(−) DLBCL (*n* = 22), and control cases [EBV(+) *n* = 8; EBV(−) *n* = 4]. EBV status for each case was determined by EBER-ISH chromogenic staining, whereby EBV(+) cases exhibited ≥50% positive nuclear staining in lesional cells, and EBV(−) cases showed no cellular staining. Representative EBV(+) and EBV(−) DLBCL cases, demonstrating EBER-ISH staining patterns, are shown in Figure 1.

The clinicopathologic characteristics of the EBV(+)/(−) DLBCL cohort (*n* = 31) are summarized in Table 1. Most patients (58%) were aged >65 years old and roughly one-third had a defined immunodeficiency, with post-transplantation (22%) and therapy-related/iatrogenic (22%) immunodeficiency states particularly enriched in EBV(+) DLBCL cases. The majority of cases (55%) were treated with standard R-CHOP chemotherapy. Less than half of cases responded to therapy, with disease-related mortality occurring in 67% of EBV(+) DLBCL cases compared to 32% of EBV(−) DLBCL cases. All EBV(+) DLBCL cases were characterized by latency status. Most cases (55%) harbored a latency III state with dual expression of LMP-1 and EBNA-2, roughly one-third of cases (33%) showed latency II state with expression of LMP-1 and lack of EBNA-2, and a single case (11%) exhibited latency I state with lack of both LMP-1 and EBNA-2 expression.

### 3.2. Global DNA Methylation Analysis of Clinical Cohort

Global DNA methylation analysis was performed on the EBV(+)/(−) DLBCL cohort (*n* = 31) and controls (*n* = 12) using the Ilumina EPIC methylation array, which interrogates approximately 950,000 CpG loci across the human genome. Quality control was carried out using the SeSAMe package to ensure data reliability and minimize technical artifacts [17]. Based on signal intensity and detection *p*-value assessments, samples and probes failing established thresholds were removed (Table S1). Probes were filtered out if they had detection *p*-values >0.05 or were poorly designed (Figure S1). Following quality control and filtering, 889,341 probes were retained for analysis.

### 3.3. Methylome Patterns: All DLBCL Versus Control Cases

**Figure 2:**
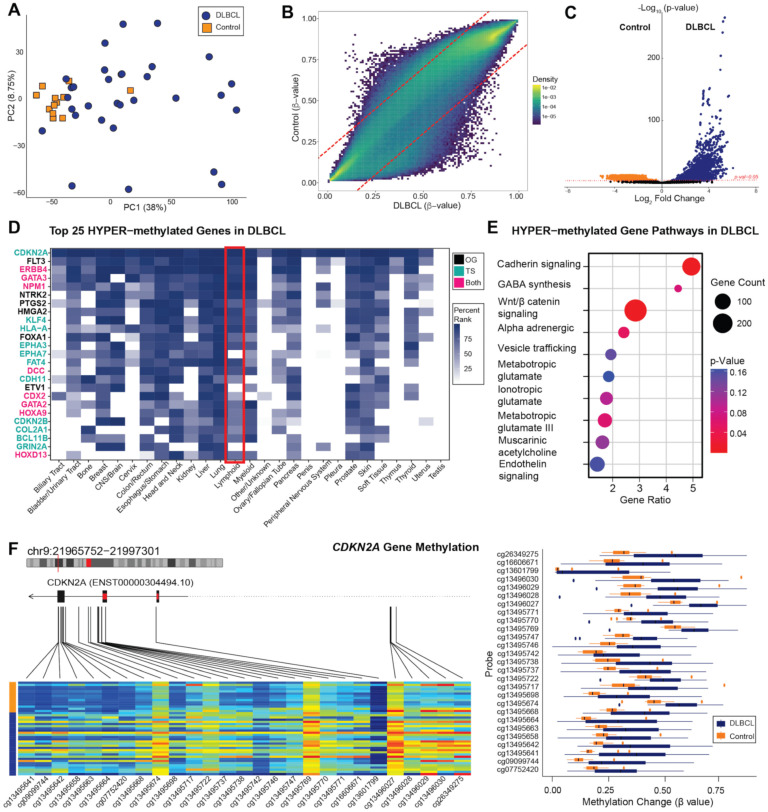
Differential methylation in all DLBCL compared to control cases. (**A**) Principal component analysis (PCA) of all EBV(+)/(−) DLBCL (*n* = 31, blue) versus all EBV(+)/(−) control cases (*n* = 12, orange) across 330,872 CpG sites, showing distinct separation of tumors from controls, using Fisher’s exact test. (**B**) Beta density scatter plot showing average differences in methylation between DLBCL (x-axis) and control cases (y-axis) across all probe sites. Red lines intersect at 0.2 and −0.2, with probes falling above or below the lines indicating an absolute difference of 0.2. Overall, DLBCL exhibit a distinct population of probes that are hypomethylated compared to control cases. (**C**) Volcano plot comparing methylation between DLBCL and controls with colors based on *p*-values (*p* = 0.05) and log-fold change highlighting significantly differentially methylated genes. (**D**) Heatmap of genes hypermethylated in DLBCL compared to controls, annotated for associations with human cancers using oncoEnrichR analysis. Hypermethylated genes highly represented in hematolymphoid malignancies (red box) include tumor suppressor genes (cyan) *CDKN2A*, *HLA-A*, and *FAT4*. Hypomethylated genes were uninformative. (**E**) Pathway enrichment analysis showing cancer-related pathways, including Wnt/β catenin and Cadherin signaling, that are hypermethylated in DLBCL compared to controls. Dot size represents number of genes involved in each pathway, and red dot color signifies statistical significance (*p* < 0.05). (**F**) Probe-level methylation map of tumor suppressor gene *CDKN2A*, showing specific CpG sites hypermethylated in DLBCL relative to controls. The heatmap y-axis shows a colored bar delineating control (orange) from DLBCL (blue) cases. OG—oncogene (black); TS—tumor suppressor (cyan); both—both TS/OG functions (magenta).

All EBV(+)/(−) DLBCL cases (*n* = 31) were directly compared to control cases (*n* = 12), which included 2 EBV(+) polymorphic lymphoproliferative disorders, 2 EBV(+) plasmacytic hyperplasia post-transplant lymphoproliferative disorders, 4 EBV(+) infectious mononucleosis tonsil/lymph nodes, and 4 EBV(−) reactive follicular hyperplasia lymph nodes. PCA revealed a clear separation between DLBCL and controls (Figure 2A). A comparison of the Beta values representing methylation levels of all probes showed an overall trend of hypomethylation in DLBCL compared to control cases (Figure 2B), suggesting global methylation associated with DLBCL pathogenesis. 

Differential methylation analysis, performed using the statistical probe filtering and normalization pipeline of the SeSAMe package [17], identified 330,872 differentially methylated CpG sites (*p* <0.05) between DLBCL and controls (Table S1; Figure S2A). Gene-level aggregation of probes identified 4110 hypermethylated genes and 120 hypomethylated genes in DLBCL (*p* < 0.05 and log_2_ fold change of 2; Table S2A), consistent with prior reports of global hypomethylation in cancer (Figure 2C) [18,19,20,21]. Many of these genes, including tumor suppressors such as *CDKN2A*, *HLA-A*, and *FAT4*, have established roles in hematolymphoid malignancies (Figure 2D). Pathway enrichment analysis of hypermethylated genes revealed significant involvement of cancer-related pathways, notably Wnt/β-catenin and Cadherin signaling, which have complex roles in lymphomagenesis (Figure 2E) [22,23,24,25,26,27]. No significant enrichment pertaining to cancer-associated pathways was discovered in the hypo-methylated gene set. The tumor suppressor gene *CDKN2A*, known to contribute to aggressive disease behavior when inactivated, [28,29,30,31,32,33] was significantly hypermethylated in DLBCL compared to controls (Figure 2F).

### 3.4. Methylome Patterns: EBV(+) DLBCL Versus EBV(−) DLBCL

**Figure 3:**
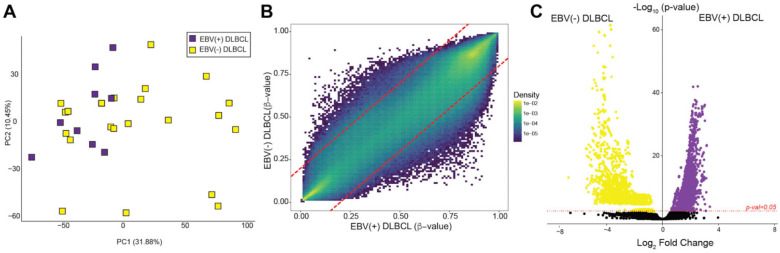
Differential methylation patterns in EBV(+) DLBCL versus EBV(−) DLBCL. (**A**) PCA of EBV(+) DLBCL (*n* = 9, purple) versus EBV(−) DLBCL (*n* = 22, yellow) showing distinct clustering of EBV(+) from EBV(−) tumors using Fisher’s exact test. (**B**) Beta density scatter plot showing average methylation differences between EBV(+) DLBCL (x-axis) and EBV(−) DLBCL (y-axis) across all probe sites, with a distinct population of probes hypermethylated in EBV(+) DLBCL. Red lines indicate significant methylation differences between the compared groups (absolute difference of 0.2). (**C**) Volcano plot comparing significantly differentially methylated genes between EBV(+) DLBCL (purple) and EBV(−) DLBCL (yellow). Colors are based on *p*-values (*p* = 0.05) and log_2_ fold change thresholds.

We next sought to identify methylation differences between EBV(+) and EBV(−) DLBCL. PCA analysis revealed clear separation between EBV(+) (*n* = 9) and EBV(−) cases (*n* = 22) (Figure 3A). Differential methylation analysis using SeSAMe identified 117,334 differentially methylated CpG sites (*p* < 0.05) between EBV(+) DLBCL and EBV(−) DLBCL (Table S1; Figure S2B). Comparison of beta values across all probes indicated an overall hypermethylation pattern in EBV(+) DLBCL relative to EBV(−) DLBCL (Figure 3B). Gene-level methylation analysis identified 2114 differentially methylated genes between EBV(+) DLBCL and EBV(−) DLBCL (*p* < 0.05 and log_2_ fold change of 2; Table S2B; Figure 3C). The distribution was skewed towards hypomethylation in EBV(+) cases (630 hypermethylated vs. 1484 hypomethylated genes). Of these, 1557 genes had cancer-related functions, with a similar skew towards hypomethylation (367 hypermethylated and 1190 hypomethylated). 

**Figure 4:**
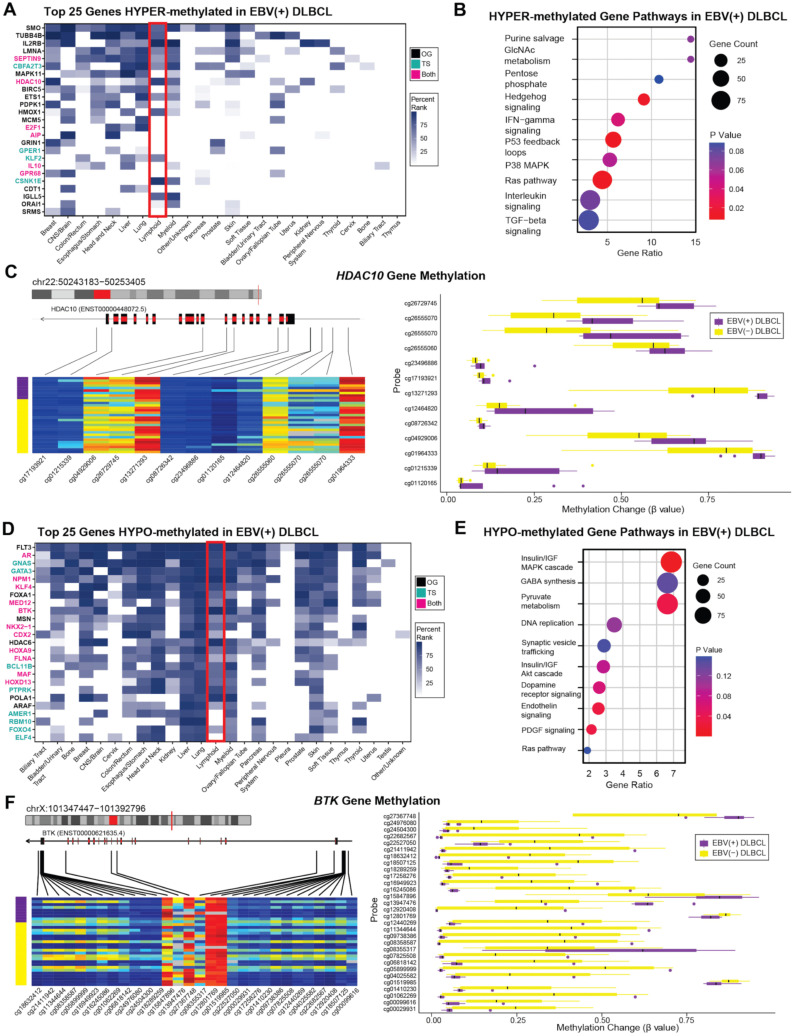
Methylation states of top genes and gene pathways in EBV(+) DLBCL versus EBV(−) DLBCL. (**A**) Heatmap of genes hypermethylated in EBV(+) DLBCL relative to EBV(−) DLBCL, annotated for associations with human cancers using oncoEnrichR analysis. The red box highlights genes significantly represented in hematolymphoid malignancies, including dual-function genes (magenta) such as *HDAC10* and only tumor suppressor function (cyan) such as *CBFA2T3* and *CSNK1E*. (**B**) Pathway enrichment analysis of hypermethylated genes in EBV(+) DLBCL. Dot size represents number of genes involved in each pathway, and red dot color signifies statistical significance (*p* < 0.05). (**C**) Probe-level methylation map of *HDAC10* gene, a gene with both tumor suppressor and oncogenic roles, showing significant hypermethylation in EBV(+) DLBCL. (**D**) Heatmap of genes hypomethylated in EBV(+) DLBCL compared to EBV(−) DLBCL. The red box highlights genes significantly associated with hematolymphoid malignancies, including dual-function genes (*BTK* and *NPM1*) and oncogenes (*FLT3* and *HDAC6*). (**E**) Pathway enrichment analysis of hypomethylated genes in EBV(+) DLBCL. (**F**) Probe-level methylation map of *BTK*, hypomethylated in EBV(+) DLBCL relative to EBV(−) DLBCL. *BTK* exhibits both tumor suppressive and oncogenic functions and is strongly associated with B-cell lymphomagenesis. OG—oncogene; TS—tumor suppressor; Both—both TS and OG functions.

Among the hypermethylated genes were both lymphoid oncogenes and tumor suppressor genes, including 15 oncogenes, 4 tumor suppressor genes, and 6 with dual oncogenic and tumor suppressor roles (Figure 4A). Notably, hypermethylated tumor suppressor genes in EBV(+) DLBCL included *CBFA2T3*, *CSNK1E*, and *HDAC10*, all of which are associated with hematolymphoid malignancies [34,35,36,37,38]. Pathway enrichment analysis of the hypermethylated genes revealed involvement in tumor suppressor pathways such as P53 feedback loops and TGF-beta signaling [2,39,40,41,42] oncogenic pathways including P38 MAPK and Ras pathways [43] and immune signaling pathways like IFN-gamma signaling [44,45] (Figure 4B). The *HDAC10* gene showed significant hypermethylation in EBV(+) DLBCL (Figure 4C). While *HDAC10* gene can function as either an oncogene or tumor suppressor depending on cellular context, it has been reported to exert tumor-suppressive effects in B-cell lymphomagenesis [38,46,47,48].

We also characterized hypomethylated genes in EBV(+) DLBCL. The top 25 hypomethylated genes included 6 oncogenes, 8 tumor suppressor genes, and 11 genes with dual functions (Figure 4D). These included oncogenes *FLT3* and *HDAC6,* as well as dual-function genes *NPM1* and *BTK*, all implicated in lymphomagenesis [49,50,51,52,53,54,55]. Pathway analysis showed that hypomethylated genes were enriched in oncogenic cascades such as the insulin/IGF MAPK cascade, DNA replication, and the RAS pathway (Figure 4E) [43,56,57]. The *BTK* gene, a well-established oncogene in B-cell lymphomagenesis, exhibited pronounced hypomethylation in EBV(+) DLBCL compared to EBV(−) DLBCL (Figure 4F) [58].

### 3.5. Immune Cell Composition in EBV(+) DLBCL

**Figure 5:**
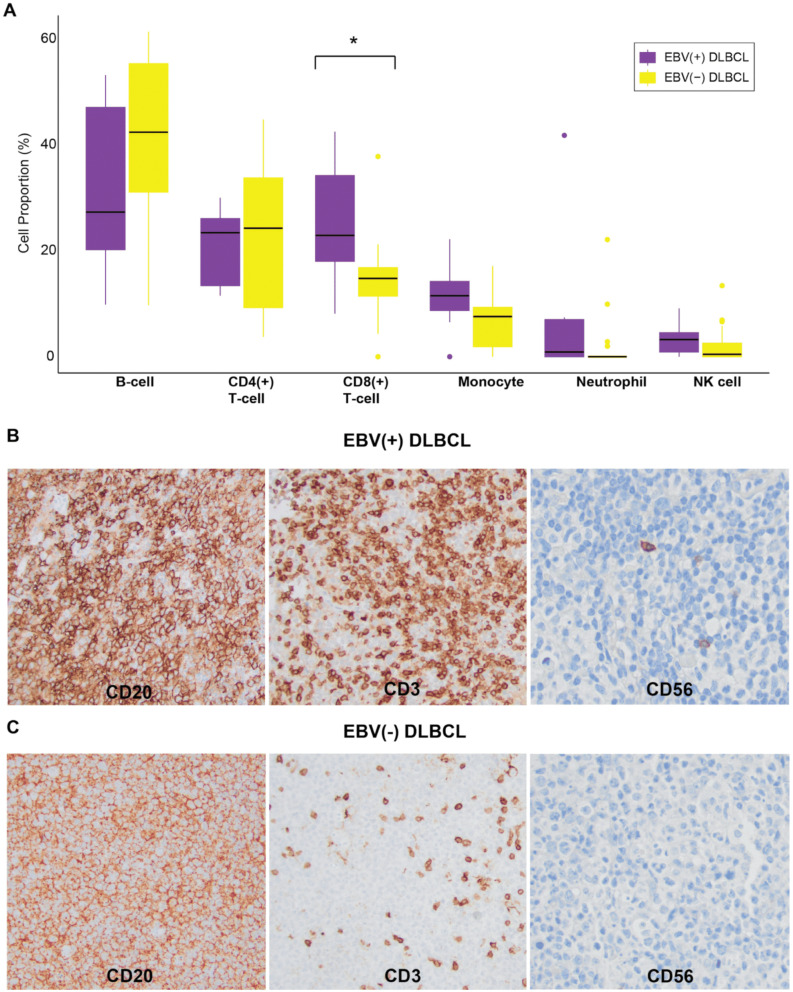
Immune cell composition of EBV(+) versus EBV(−) DLBCL. (**A**) Immune cell type inference revealed enrichment of CD8(+) T-cells (*p* = 0.01), monocytes (*p* = 0.11), neutrophils (*p* = 0.08), and NK cells (*p* = 0.11) in EBV(+) DLBCL. In contrast, EBV(−) DLBCL showed an enrichment of B-cells (*p* = 0.07) and CD4(+) T-cells (*p* = 0.63) relative to EBV(+) tumors. Statistical comparisons were performed using the Wilcoxon test. (**B**) Immunohistochemistry of EBV(+) DLBCL showing an inflammatory microenvironment enriched in CD3(+) T cells (20×) and CD56(+) NK cells (40×), with reduced CD20(+) B-cells (20×). (**C**) Immunohistochemistry of EBV(−) DLBCL showing enrichment of CD20(+) B-cells (20×), with relatively fewer CD3(+) T cells (20×) and absence of CD56(+) NK cells (40×). Varying magnification was used to demonstrate the overall staining pattern in B-cells and T-cells (20×) and individual cellular staining in NK cells (40×). The asterisk indicates statistical significance (*p* < 0.05).

The immune cell composition of DLBCL plays an important role in the clinicopathologic progression of the disease. To assess differences between EBV(+) and EBV(−) DLBCL, we performed immune cell inference through deconvolution of the methylation data. This analysis revealed a distinct immune cell composition profile for EBV(+) DLBCL, with an enrichment of certain immune cell types and relative depletion of others. Specifically, EBV(+) DLBCL demonstrated a significant predominance of CD8(+) T-cells (*p* = 0.01), with a relative predominance of monocytes (*p* = 0.11), neutrophils (*p* = 0.08), and NK cells (*p* = 0.11). In contrast, B-cells (*p* = 0.07) and CD4(+) T cells (*p* = 0.63) were relatively depleted compared to EBV(−) DLBCL (Figure 5A). These findings were supported by immunohistochemical staining for B-cells (CD20), T cells (CD3), and NK cells (CD56) in representative cases of EBV(+) and EBV(−) DLBCL (Figure 5B and 5C, respectively). EBV(+) tumors showed an inflammatory background enriched in CD8(+) T cells and NK cells, while EBV(−) cases exhibited higher B-cell content.

## 4. Discussion

EBV(+) DLBCL is more clinically aggressive, resulting in worse overall survival and treatment response compared to EBV(−) disease [1,2,3]. However, there is no targeted therapy or prognostic biomarker for EBV(+) DLBCL. Few studies have investigated the epigenomic heterogeneity of EBV-mediated DLBCL, with most studies focused heavily on overall DLBCL pathogenesis and non-viral disease mechanisms [59,60,61,62,63]. The epigenetic landscape of B-cell lymphomas is increasingly being recognized as crucial in disease pathogenesis and therapeutic targeting [64,65,66,67]. EBV epigenetically modifies host cellular genome through selective DNA methylation, potentially promoting lymphomagenesis [10,11]. To better understand the influence of EBV on the DNA methylome of DLBCL, this study provides a systematic epigenetic characterization of EBV(+) DLBCL in relation to EBV(−) DLBCL and controls. Our results indicate that EBV(+) DLBCL exhibits a distinct DNA methylome from EBV(−) DLBCL, possibly influencing the observed clinical heterogeneity in EBV(+) tumors while also revealing therapeutic and prognostic biomarkers of disease.

A total of 2114 differentially methylated genes, corresponding to 1557 cancer-associated genes, were identified in EBV(+) DLBCL compared to EBV(−) DLBCL, defining a biologically distinct DNA methylome. This trend was also observed in the DLBCL-versus-controls analysis, where DLBCL cases exhibited global hypomethylation across all probe sites relative to controls. Global DNA hypomethylation is a well-documented feature of tumors compared to normal tissue and is considered a driver of tumorigenesis by enabling transcriptional activation of oncogene promoters, promoting genomic instability, and increasing tumor heterogeneity [19]. In DLBCL specifically, hypomethylation relative to normal B-cells has been reported and can serve as both a diagnostic and prognostic biomarker, capable of distinguishing molecular subtypes [21]. A study by Chambwe et al. demonstrated that increased DNA methylation variability in DLBCL correlates with worse clinical outcomes and overall survival. This variability reflects a loss of characteristic bimodal methylation distribution seen in normal tissue, a phenomenon observed across multiple tumor types [21,68]. Such epigenetic variability is closely linked to clinical outcome and tumor heterogeneity as part of tumorigenesis [68]. Our findings confirm the presence of epigenetic variability in all DLBCL cases relative to control tissue, as well as within EBV-defined subgroups, supporting the utility of DNA methylation patterns as a global biomarker of tumor heterogeneity.

While the genes in EBV(+) DLBCL were skewed towards hypomethylation, the overall methylome across all probe sites exhibited a hypermethylated state in EBV(+) compared to EBV(−) tumors. Furthermore, although the global methylome in DLBCL showed a hypomethylation bias toward controls, the subset of genes of oncologic significance was predominantly hypermethylated, with no significant hypomethylated genes detected in this group. These findings are consistent with several studies demonstrating EBV’s role in promoting global hypermethylation of the host genome during carcinogenesis [10,69]. Both in vitro and clinical studies have shown that EBV actively modifies its own genome and the host cellular genome to establish viral latency, evade immune surveillance, and persist in host B-cells [10,11,64,69]. EBV-mediated lymphoblastoid transformation is thought to result from accumulated pathogenic mutations and epigenetic modifications that drive oncogenesis. Hypermethylation of promoter regions in key tumor suppressor genes, coupled with hypomethylation of oncogenes, has been cited as a critical epigenetic mechanism in lymphomagenesis [11,64]. In DLBCL, such differential methylation patterns can define molecular subtypes and, in some cases, dictate prognosis [21,63,65,70]. Our findings demonstrate that EBV-mediated DLBCL harbors both hyper- and hypomethylated states within its tumor methylome, suggesting potential drivers of aggressive lymphomagenesis compared to EBV(−) disease.

We have characterized distinct methylomes defining DLBCL from control tissue and EBV(+) from EBV(−) tumors. The differential methylation of oncogenic genes in DLBCL, overall, skewed towards a hypermethylated state. However, we have also identified a significant set of hypomethylated genes in EBV(+) DLBCL as well. These patterns support distinct tumor heterogeneity and differences in prognosis in viral-mediated DLBCL. Hypermethylated genes of DLBCL included both oncogenic and tumor suppressor genes, with several exhibiting both functions. Overall, however, there were more tumor suppressor genes that were hypermethylated in DLBCL relative to controls. For EBV(+) DLBCL, we identified a similar mix of oncogenes, tumor suppressor genes, and genes with both functions that were hypermethylated. The hypomethylated genes also exhibited a mix of oncologic function with a predominance of genes with both oncogenic and tumor suppressor functions. All genes identified across the compared groups correlated to significant oncologic function in hematolymphoid malignancy and other human cancers. These defining genetic patterns correlated with unique gene pathways. DLBCL showed enrichment for hypomethylated genes involved in Wnt/β-catenin and cadherin signal transduction pathways, which are known to play complex roles in lymphomagenesis [22,23,24,25,26,27]. Upregulation of Wnt/β-catenin signaling can cause more aggressive clinical behavior in DLBCL by promoting the endothelial to mesenchymal transition [25,26]. The tumor suppressor gene *CDKN2A* was significantly hypermethylated in DLBCL relative to controls, suggesting downregulation and loss of function. The functional loss of *CDKN2A* is linked to dysregulated P53 expression leading to more aggressive lymphomagenesis and worse clinical outcome in DLBCL, despite standard chemotherapy [28,29,30,31,32,33].

Both hypermethylated and hypomethylated oncologic genes were detected in EBV(+) DLBCL relative to EBV(−) tumors, reflecting the tumor heterogeneity of viral-mediated disease. Hypermethylated oncologic genes correlated to tumor suppressive genetic pathways including P53 feedback loops and TGF-beta signaling. Loss of normal P53 function is associated with DLBCL occurrence and worse overall prognosis, even in the setting of standard R-CHOP chemotherapy [28,39]. Transforming growth factor beta (TGF-beta) signaling is highly conserved and important for organismal development, but has been shown to be exploited by various human cancer types to promote tumorigenesis, immune system evasion, and microenvironment modification [71]. In its normal state, TGF-beta functions as a tumor suppressor in B-cells, promoting cryostasis and modulating cellular differentiation and apoptotic pathways [42,71]. However, in vitro studies have shown that EBV-infected B-cell lymphoma cell lines dysregulate TGF-beta receptor signaling, making the cells impervious to the anti-tumor effects of endogenous TGF-beta [40,72]. Further, these studies linked the dysregulation of TGF-beta signaling with constitutive activation of the P38 MAPK oncogenic pathway, which we also found to be enriched in the hypermethylated gene set of EBV(+) DLBCL [40]. Interestingly, the IFN-gamma pathway was also enriched, suggesting functional suppression of its immunogenic anti-viral effects in EBV-mediated lymphomagenesis [45].

Finally, we highlighted the *HDAC10* gene as significantly hypermethylated and potentially suppressed in EBV(+) DLBCL relative to EBV(−) disease. The *HDAC10* gene encodes histone deacetylase 10, a class II family, which has crucial roles in epigenetic regulation. Depending on cellular context, *HDAC10* can induce both oncogenic and tumor suppressive effects across an array of human cancer types. In B-cell lymphoma, it can exert tumor suppressive effects in concert with the dysregulation of the TGF-beta pathway [38,73]. The hypermethylation of this gene in EBV(+) DLBCL implies suppression of the *HDAC10* pathway and loss of inherent tumor suppressive function.

Several pathways were identified to be significantly enriched in the hypomethylated gene set in EBV(+) DLBCL compared to EBV(−) DLBCL. These pathways included prominent oncogenic signaling like the MAPK cascade, DNA replication, and RAS pathway, which are highly represented across human cancer types [43,56,57]. EBV-latent proteins, which define EBV latency states, can promote oncogenesis by epigenetically altering DNA replication and maintenance pathways [74,75]. In our study, the viral latency states of EBV(+) DLBCL were characterized by IHC expression of EBV latency proteins LMP-1 (latent member protein-1) and EBNA-2 (EBV nuclear antigen-2). LMP-1, in particular, is an essential oncoprotein in facilitating B-cell transformation and has been proven to upregulate expression of DNA methyltransferase 1 (DNMT1) and 3 a/b (DNMT3a/b), an essential component of the DNA replication/maintenance pathway. DNMT upregulation activates promoter hypermethylation of key tumor suppressor genes, supporting B-cell transformation and oncogenesis [75]. In our cohort, the majority of EBV(+) DLBCL (89%) exhibited expression of LMP-1, with enrichment of latency II and III states, possibly linking the oncogenic activity of LMP-1 with the observed DNA replication signaling pathway in EBV(+) DLBCL.

In addition, the known oncogenes *FLT3*, *HDAC6*, *NPM1*, and *BTK* were significantly hypomethylated in EBV(+) DLBCL, suggesting activation [49,50,51,52,53,54,55]. In particular, Bruton’s tyrosine kinase (*BTK*) is highlighted as significantly hypomethylated in EBV(+) DLBCL. BTK is a well-characterized and important non-receptor tyrosine kinase that functions in the B-cell signaling pathway. Abnormal upregulation of BTK can turn on its oncogenic function in promoting B-cell proliferation, activation, survival, and differentiation [54,58]. BTK inhibitors are powerful targeted therapies used in an array of B-cell malignancies, including DLBCL [53,55]. BTK activates a signaling cascade which involves the RAS pathway and downstream transcription initiation of the *MYC* oncogene [54].

The immune cell microenvironment of DLBCL plays a critical role in tumor aggression and response to therapy. Inference of immune cell composition in EBV(+)/(−) DLBCL was made using global DNA methylation data. EBV(+) DLBCL exhibited a significant predominance of cytotoxic CD8(+) T-cells relative to EBV(−) DLBCL (*p* = 0.01), as well as a relative predominance in neutrophils, monocytes, and NK cells comprising the background inflammatory microenvironment. In contrast, EBV(−) DLBCL showed a relative predominance in B-cells and CD4(+) T-cells. These findings were confirmed using immunohistochemical stains for B-cells (CD20), T-cells (CD3), and NK cells (CD56). This unique tumor microenvironment composition of EBV(+) DLBCL with a relative increase in inflammatory cells, CD8(+) T-cells, and reduced B-cells has been confirmed by multiple studies [76,77,78]. The “immune-rich” environment of EBV(+) DLBCL has been observed across immunodeficiency states and is associated with the activated B-cell (ABC) phenotype. The cellular composition includes immunologically exhausted cytotoxic T-cells and NK cells and the upregulation of immune checkpoint proteins (PD-L1) and associated monocytes/macrophages [76,77]. This constellation of factors all lead to increased tumor heterogeneity, an immunosuppressive profile, and worse clinical prognosis in EBV(+) DLBCL across various states of immunodeficiency [76,78].

While this study represents a novel characterization of EBV-mediated DLBCL using global methylation analysis, there are several limitations that should be noted. First, while this is a single-institutional pilot study, the cohort size of EBV(+) DLBCL is small (*n* = 9), limiting comparability of the findings with EBV(−) tumors. Second, the nature of DLBCL as a systemic disease with limited clinical sampling of diagnostic tissue specimens makes obtainment of control tissue from the same patient very difficult in this retrospective approach. In turn, we obtained EBV(+)/(−) normal lymph node/tonsillar tissue as well as comparison cases of other EBV(+) B-cell lymphoproliferative disorders. We acknowledge that the control tissue from different patients limits comparability of the DLBCL findings. Lastly, the global EPIC methylation array employed by this study, although comprehensive, has its limitations in understanding more granular nuances of epigenetic change between compared datasets. Our study is a pilot initiative with the intent to first characterize the overall methylome of EBV-mediated DLBCL. Future, more extensive studies are necessary on a larger clinical cohort utilizing an integrative analysis of epigenetic and whole transcriptomic approaches. This will help elucidate specific genetic pathways and epigenetic alterations that may lead to diagnostic and prognostic biomarker identification in EBV(+) DLBCL.

## 5. Conclusions

Using a global DNA methylation array, we found that the methylome of EBV(+) DLBCL across immunodeficiency states is epigenetically distinct from EBV(−) disease, and that DLBCL as a whole is epigenetically distinct from control tissue cases. Both EBV(+) DLBCL and all DLBCL cases exhibited distinct patterns of hyper- and hypomethylation, which corresponded to specific gene pathways with complex roles in lymphomagenesis. EBV(+) DLBCL demonstrated greater hypermethylation across all probe sets compared to EBV(−) tumors, with enrichment of hypermethylated genes involved in tumor-suppressive pathways such as P53 feedback loops and TGF-beta signaling. The immune cell microenvironment of EBV(+) DLBCL, inferred from methylation data, displayed an immune-rich yet immunosuppressive profile, characterized by increased cytotoxic T cells and NK cells relative to the neoplastic population. These findings support the conclusion that EBV(+) DLBCL, across various immunodeficiency settings, harbors an epigenetically distinct methylome that may contribute to the observed tumor heterogeneity and clinical aggressiveness of this disease. Larger studies are warranted to more comprehensively characterize the methylation landscape of EBV-mediated DLBCL, with the goal to identify targetable diagnostic and prognostic biomarkers.

## Figures and Tables

**Table 1:** Clinicopathologic and immunophenotypic characteristics of EBV(+) and EBV(−) DLBCL (*n* = 31). Not included are EBV(+)/(−) control cases (*n* = 12). * Therapy-related/iatrogenic immunodeficiency includes post-cytotoxic chemotherapy/radiation therapy for prior malignancy, either concurrently or within 15 years prior to DLBCL. ** Autoimmune disease immunodeficiency includes anti-TNF/methotrexate and systemic corticosteroid therapy, either concurrently or within 15 years prior to DLBCL. R-CHOP—Rituximab, Cyclophosphamide, Doxorubicin Hydrochloride, Vincristine Sulfate, Prednisone.

Clinicopathologic Characteristics	Total Cohort (n = 31)	EBV(+) DLBCL (n = 9)	EBV(−) DLBCL (n = 22)
Sex (male/female)	18/13	8/1	10/12
Age, mean, year (range)	67 (26–90)	68 (27–90)	65 (26–90)
Older than 65	18 (58%)	6 (67%)	12 (55%)
Lymph Nodal Lesion	19 (61%)	4 (44%)	15 (68%)
Extra-nodal Lesion	12 (39%)	5 (56%)	7 (32%)
Defined Immunodeficiency	10 (32%)	5 (56%)	5 (23%)
Post-transplantation (solid organ/stem cell)	3 (10%)	2 (22%)	1 (5%)
Therapy-related/Iatrogenic *	7 (23%)	2 (22%)	5 (23%)
Autoimmune disease **	2 (6%)	1 (11%)	1 (5%)
Primary immunodeficiency	1 (3%)	1 (11%)	0
Multiple immunodeficiencies	3 (10%)	1 (11%)	2 (9%)
No Defined Immunodeficiency	21 (68%)	4 (44%)	17 (77%)
Presumed immunosenescence	11 (35%)	3 (33%)	8 (36%)
Treatment			
None or localized radiation only	4 (13%)	1 (11%)	3 (14%)
Chemotherapy (R-CHOP)	17 (55%)	5 (56%)	12 (55%)
Chemotherapy other than R-CHOP	6 (19%)	2 (22%)	4 (19%)
Unknown	4 (13%)	1 (11%)	3 (14%)
Response to Treatment			
Complete response	14 (45%)	2 (22%)	12 (55%)
Death due to disease	13 (42%)	6 (67%)	7 (32%)
Unknown	4 (13%)	1 (11%)	3 (14%)
DLBCL Subtype			
Activated B-cell (ABC)	19 (61%)	8 (89%)	11 (50%)
Germinal center B-cell (GCB)	12 (39%)	1 (11%)	11 (50%)
EBV Latency Pattern			
Latency I: LMP1(−), EBNA2(−)		1 (11%)	
Latency II: LMP1(+), EBNA2(−)		3 (33%)	
Latency III: LMP1(+), EBNA2(+)		5 (56%)	

## Data Availability

The original data presented in the study are openly available in GEO at https://www.ncbi.nlm.nih.gov/geo/query/acc.cgi?acc=GSE306846.

## Supplementary Materials

The following supporting information can be downloaded at https://www.mdpi.com/article/10.3390/cancers17182994/s1, Figure S1: Exclusion of low-quality samples; Figure S2: Volcano plots of total probes for group comparisons; Table S1: Group sample and probe numbers for complete dataset studied using Illumina EPIC Methylation Array; Table S2: Differentially methylated genes.
