# Supplementary material for: The EBV-Positive Tumor Methylome Is Distinct from EBV-Negative in Diffuse Large B-Cell Lymphoma
Source: Cancers (Basel). 2025 Sep 13;17(18):2994. doi: 10.3390/cancers17182994 (PMC12468171; doi:10.3390/cancers17182994)
Supplement: Supplementary file 1 [file cancers-17-02994-s001.zip › TableS1.pdf]

| Group | DLBCL vs Control | EBV(+) DLBCL vs EBV(-) DLBCL |
|---|---|---|
| **Total Samples** | 43 | 31 |
| EBV(+) DLBCL | 9 | 9 |
| EBV(-) DLBCL | 22 | 22 |
| EBV(+) Control | 8 | 0 |
| EBV(-) Control | 4 | 0 |
| **Total Probes** | 937,690 | 937,690 |
| Used | 889,341 | 874,394 |
| Significant ($p<0.05$) | 330,872 | 117,334 |

**Supplemental Table S1. Group Sample and Probe Numbers for Complete Dataset Studied using Illumina EPIC Methylation Array.**
