# Supplementary material for: The EBV-Positive Tumor Methylome Is Distinct from EBV-Negative in Diffuse Large B-Cell Lymphoma
Source: Cancers (Basel). 2025 Sep 13;17(18):2994. doi: 10.3390/cancers17182994 (PMC12468171; doi:10.3390/cancers17182994)
Supplement: Supplementary file 1 [file cancers-17-02994-s001.zip › TableS2_caption.pdf]

[See excel file]

Supplemental Table S2. Differentially Methylated Genes. A) Differentially methylated gene list for DLBCL (n=31) versus control (n=12) comparison cohort with p-values and false discovery rates (FDR). **B)** Differentially methylated gene list for EBV(+) DLBCL (n=9) compared to EBV(-) DLBCL (n=22).
