# Supplementary figures and images for: The EBV-Positive Tumor Methylome Is Distinct from EBV-Negative in Diffuse Large B-Cell Lymphoma
Source: Cancers (Basel). 2025 Sep 13;17(18):2994. doi: 10.3390/cancers17182994 (PMC12468171; doi:10.3390/cancers17182994)

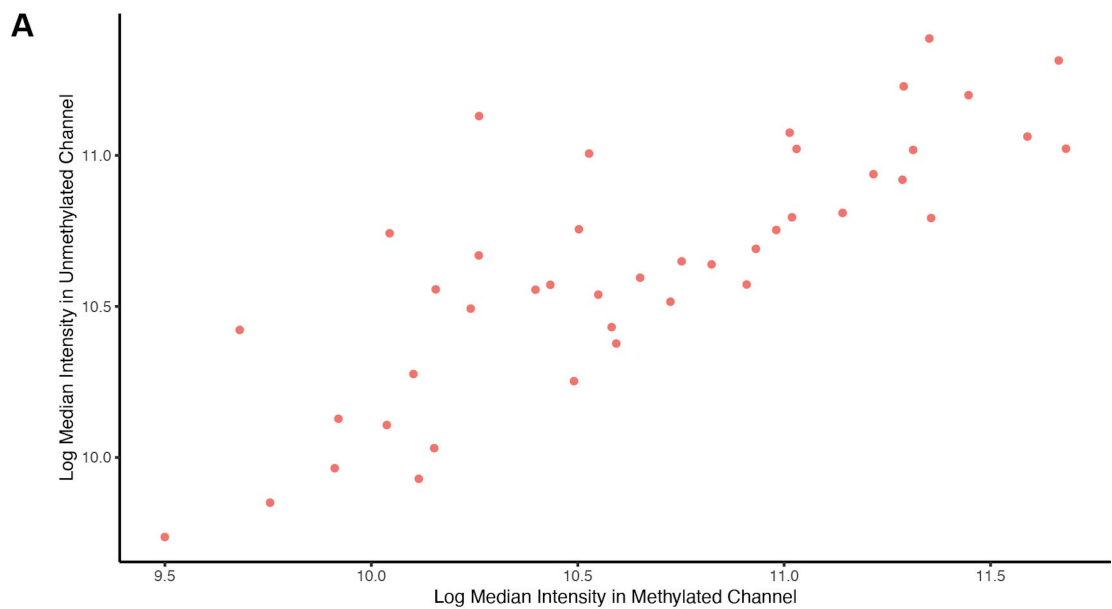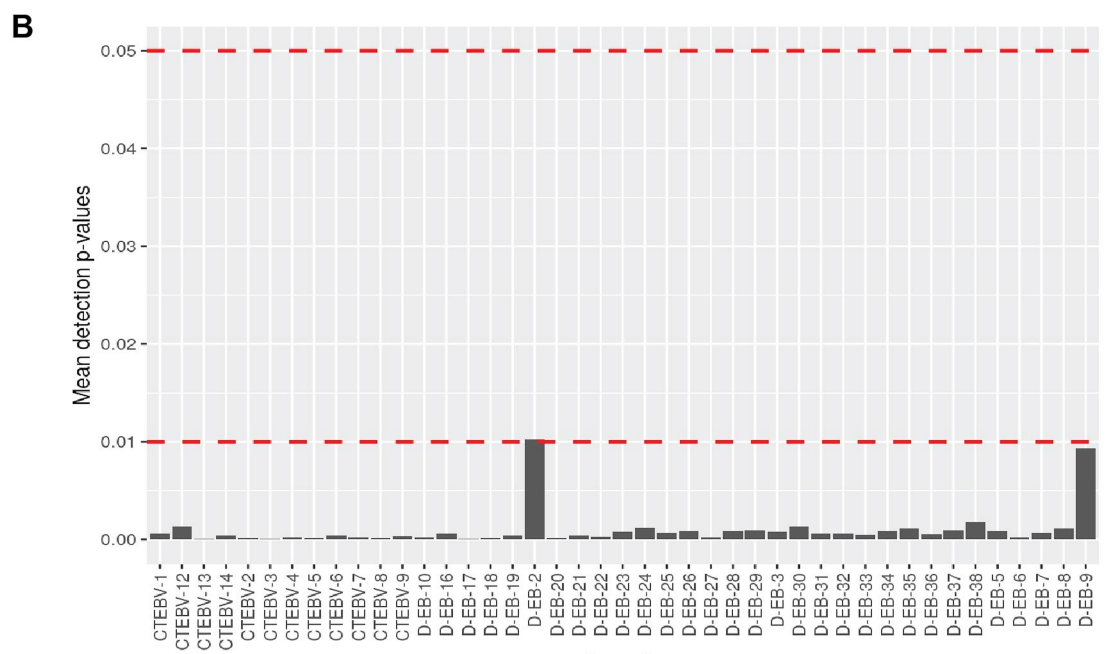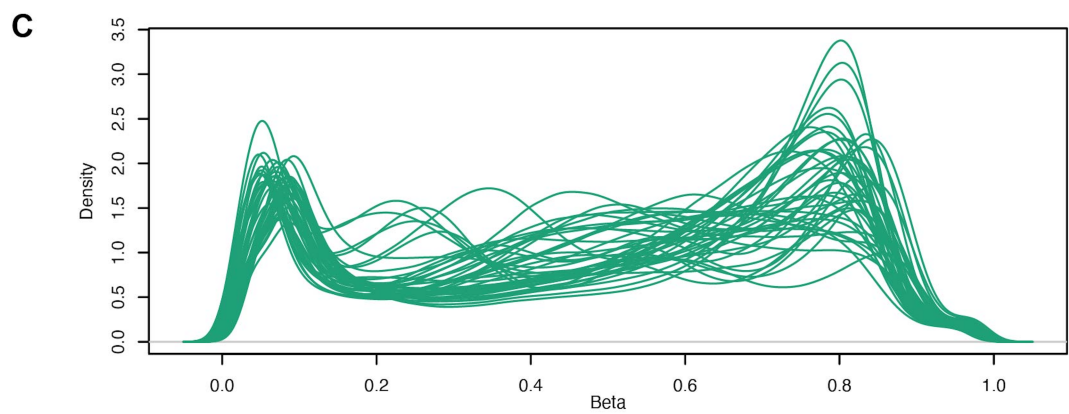

Supplement: Supplementary file 1 [file cancers-17-02994-s001.zip › FigureS1.pdf]

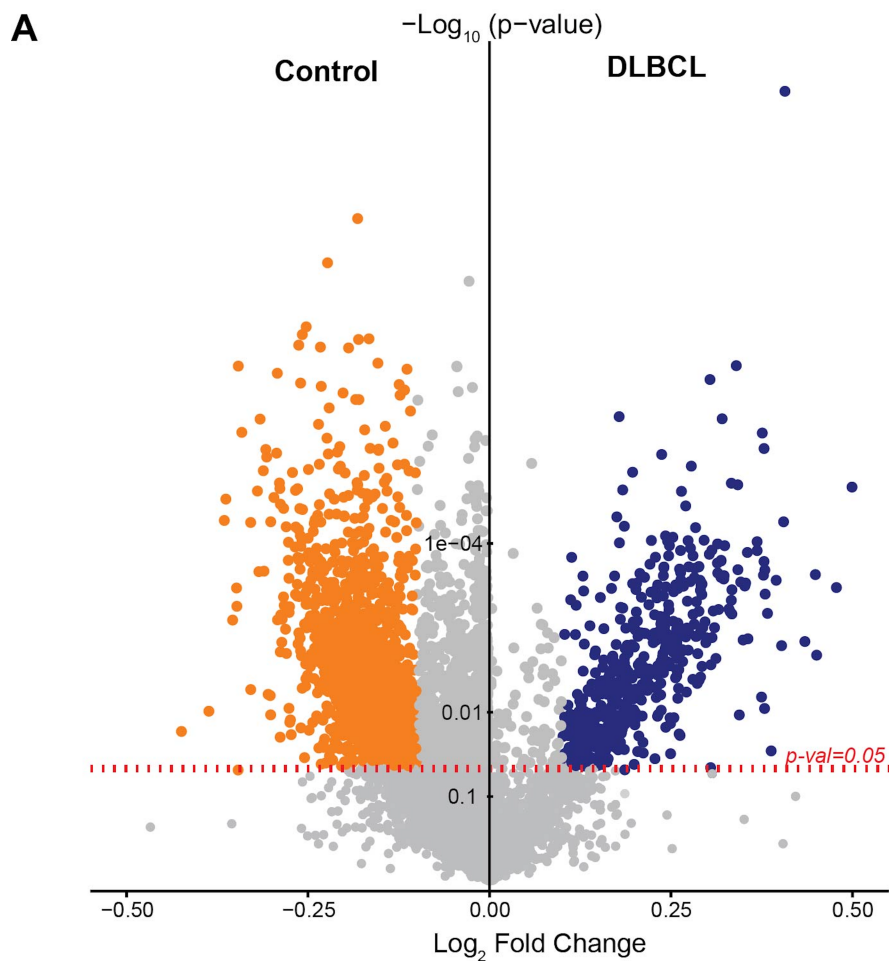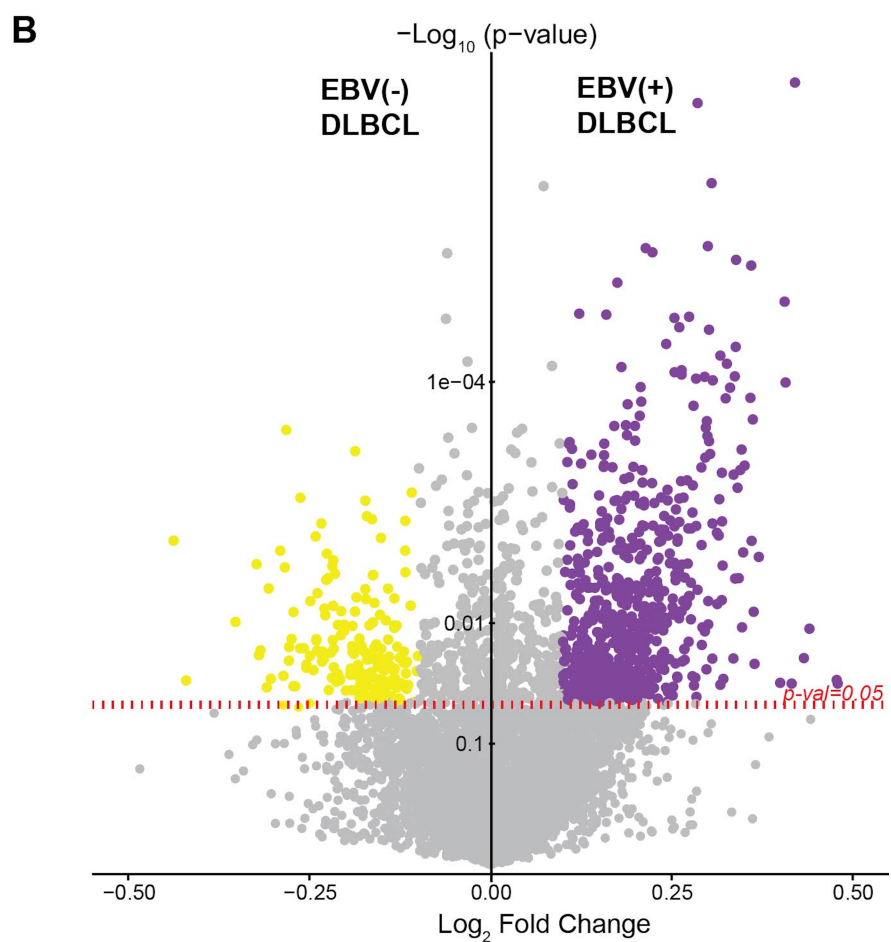

Supplement: Supplementary file 1 [file cancers-17-02994-s001.zip › FigureS2.pdf]
